# Highly electroactive Co–ZnO/GO nanocomposite: Electrochemical sensing platform for oxytetracycline determination

**DOI:** 10.1016/j.heliyon.2024.e30265

**Published:** 2024-04-26

**Authors:** Haifa Mliki, Mosaab Echabaane, Ahlem Rouis, Jaber Mohamed El Ghoul, Francois Bessueille, Dhekra Ayed, Nicole Jaffrezic-Renault

**Affiliations:** a Laboratory of Interfaces and Advanced Materials (LIMA) Faculty of Sciences of Monastir, University of Monastir, 5019, Monastir, Tunisia; b CRMN, Centre for Research on Microelectronics and Nanotechnology of Sousse, NANOMISENE, LR16CRMN01, 4034, Sousse, Tunisia; c Laboratory of Physics of Materials and Nanomaterials Applied at Environment (LaPhyMNE), Gabes University, Faculty of Sciences in Gabes, 6072, Tunisia; d University of Lyon, Institute of Analytical Sciences, UMR-CNRS 5280, 5, La Doua Street, Villeurbanne, 69100, France

**Keywords:** Electrochemical sensors, Cobalt doped zinc oxide/graphene oxide nanocomposite, Oxytetracycline detection

## Abstract

Antimicrobial residues in animal-derived foods have become a major source of concern around the world. Oxytetracycline (OTC), one of these antibiotics that belongs to the tetracycline family should be detected in these matrices. Nanostructured metal oxides have attracted a lot of scientific attention due to their special characteristics that can be exploited for creating innovative nanodevices. Therefore, in the present study, we report the fabrication of cobalt-doped ZnO/GO nanocomposites for OTC sensors using a simple and environmentally friendly method that does not require toxic solvents. Contact angle measurements, X-ray diffraction (XRD), Fourier transform infrared spectroscopy (FTIR), scanning electron microscopy (SEM), X-ray photoelectron spectroscopy (XPS) and UV–Vis were used to confirm the successful fabrication of the Co–ZnO/GO nanocomposite and to determine the surface area, Structural, morphological features, chemical composition and purity of the nanocomposite. The electrochemical and electrocatalytic properties were recorded using cyclic voltammetry (CV), electrochemical impedance spectroscopy, and differential pulse voltammetry (DPV). Optimizing parameters such as scan rate, pH value, deposition time, and deposition potential, we achieve a wide linear concentration range from 10^−12^ M to 10^−7^ M, with an impressive detection limit of 1.6 10^−13^ M.Notably, our sensor exhibits remarkable selectivity, demonstrating its usefulness for the detection of oxytetracycline traces in real milk samples. These results emphasize the novelty and practical significance of our work and provide a promising avenue for the development of sensitive and selective electrochemical sensing platforms in various fields.

## 1 Introduction

The escalating human population has led to a significant increase in demand for sustainable food production. The antibiotic oxytetracycline and other pharmaceutical compounds play a crucial role in increasing food production by effectively combating bacterial infections in crops and livestock. These antibiotics help control a wide range of bacterial infections that can affect agricultural yields, including those caused by pathogens such as bacteria, fungi and protozoa.

[1].Consequently, the global use of oxytetracycline antibiotics has seen a remarkable upsurge since their introduction, particularly in modern agriculture and animal husbandry [2,3].

However, this widespread use has raised concerns about its potential impact on human health, agricultural sustainability and the environment [1,4].Therefore, the search for feasible and environmentally friendly solutions to mitigate the risks associated with the extensive use of oxytetracycline antibiotics has become an urgent contemporary challenge that is essential for protecting ecosystem health and preserving ecological integrity.

Several monitoring techniques have been developed for the determination of these antibiotics, including enzyme-linked immunosorbent tests (ELISA), high-performance liquid chromatography (HPLC) [5], and liquid chromatography-tandem mass spectrometry (LC-MS/MS) [6,7]. While these existing methods involve complex instrumentation setups, labor-intensive pre-treatment steps, limited accessibility, and low sensitivity [8]**,** the electrochemical method stands out as an effective approach for the detection of residual traces of antibiotics in food [9,10] due to their significant benefits, such as their simplicity, high sensitivity, reproducibility, cost effectiveness and non-destructive quantitative detection.

Carbon-based nanomaterials, particularly graphene oxide (GO), have been extensively utilized in sensor applications due to their unique properties, including good mechanical strength, high surface area, and excellent electrical conductivity. Modification of electrodes with GO and its hybrids has been shown to enhance the detection limit and range for various analytical targets [11,12]. Because GO has functional groups such as hydroxyl, epoxy, and carbonyl, which are advantages for adsorbing heavy metals and antibiotics, many nanocomposites with inert metals have been successfully used in antibiotic residue adsorption [13,14].Recently, ZnO has become a very interesting common semiconducting material. It has attracted great concern in the materials science community because of its stability, high mechanical strength, and good catalytic activity. It presents a wide band gap in metal oxide with a direct band gap energy of about 3.37 eV. It is non-toxic, and offers high thermal and electrical stability. A further important point that needs to be mentioned here is that ZnO is “Generally Recognized as Safe” by the US Food and Drug Administration [15]. Doping ZnO with transition metals like cobalt (Co) introduces additional functionalities and properties to the material. Doping doesn't only affect the band gap of metal oxide, but it also modifies the oxidation states and structural parameters of the crystal [16]. The synthesis of zinc oxide, cobalt oxide and the blend of zinc and cobalt hydroxides has recently garnered attention in the research community due to the potential to achieve desired electrical and optical characteristics [17].The incorporation of cobalt into ZnO lattices is particularly notable for improving electrocatalytic behavior, which can have implications for antibacterial applications [18].

Cobalt-doped ZnO emerges as a promising component for nanocomposite materials, offering tailored properties for specific applications. This underscores the importance of understanding and harnessing the synergistic effects of doping in semiconductor materials like ZnO for advancing various technological applications.

In the current study,we report a facile one-step chemical approach to obtain a high quality Co–ZnO/graphene oxide (Co–ZnO/GO) nanocomposite, to produce well-dispersed Co–ZnO nanoparticles *in situ* grown on the surfaces of GO using a simple ultrasonication method. Afterwards, the as-fabricated nanocomposite was used to develop an improved sensing platform for the electrochemical detection of oxytetracycline in milk sample. The developed sensor demonstrates significant catalytic ability, fast electron transfer, and excellent analytical performance for oxytetracycline detection, including a large linear range, low detection limit, and sufficient recovery rates. Our study contributes to the advancement of electrochemical sensing technology for food safety applications, particularly in the detection of antibiotic residues in dairy products. Through detailed comparisons with existing methods, we demonstrate the superiority of our developed sensor in terms of sensitivity, specificity, and ease of use.

## 2 Experimental part

### 2.1 Chemical and materials

Graphene oxide (GO) is commercially accessible in an aqueous solution with a concentration of 4 g/L, produced through a modified Hummers' method. This solution is obtained from Graphenea S.A, based in Spain. Zinc oxide (ZnO) and cobalt doped zinc oxide Co–ZnO nanoparticles (NPs) were synthesized via a sol-gel method by Dr. Jaber Mohamed El Ghoul in LaPhyMNE at Gabes University [19,20]. Acetone, ethanol, isopropanol, oxytetracycline (OTC), potassium ferricyanide (K_3_Fe(CN)_6_), K^+^, Na^+^, lysine, citric acid, sulfate copper (CuSO_4_), tryptophan, cysleine, Zn^2+^, Al^3+,^ Mg^2+,^ and ammonium acetate (CH_3_COO^−^, NH_4_^+^) were procured commercially from SIGMA-ALDRICH (France). The milk, that needed to be diluted, was taken from the supermarket. All solutions were prepared with distilled water.

### 2.2 Preparation of Co–ZnO/GO nanocomposite

We propose a simple and environmentally friendly method for the preparation of a Co–ZnO/GO nanocomposite in a deionized water solution that does not require toxic solvents and allows direct growth of nanoparticles on graphene without post-annealing or calcination. In a typical experiment, a 1 ml of 4 g/L GO solution was diluted with the same amount of deionized water (1 ml). Then 3 mg of Co–ZnO powder was added to the suspension and stirred at room temperature. The resulting solution was sonicated for 2 h. This step facilitated the dispersion and interaction of the components. For comparison purposes, ZnO/GO was prepared according to the same protocol [21].

The nanocomposites were formed due to the attraction of positively charged metal/metal oxide ions by the negatively charged polarization bonds of GO [22].

### 2.3 Fabrication of the sensor

To prepare the electrode**,** an ITO -coated glass substrate (thickness of 100 nm, sheet resistance of 20/cm^2^), purchased from Merck Display Technologies, was first ultrasonically consecutively cleaned in baths of acetone and isopropanol for 20 min and then washed with distilled water. The electrode was then dried at 60 °C for 30 min under vacuum conditions. Subsequently, 30 μL of the GO, ZnO/GO, and co-ZnO/GO suspensions were dropped onto the ITO surface and dried for 1 h at 120 °C under vacuum conditions to remove any residual solvent.

### 2.4 Instrumentation

To characterize the prepared co-ZnO/GO nanocomposite various techniques were employed such as: scanning electron microscope SEM, (a Tescan Vega SBU scanning electron microscope), operating at an accelerating voltage of 20 kV, equipped with a Bruker Esprit Compact EDS detector. The surface wettability of the films was carried out using a “Digidrop” contact angle measuring device from GBX (France) with the “Win Drop” software, which made it possible to check the hydrophobicity and adhesion of the deposited film.

Additionally, X-ray diffraction technique (XRD) (Bruker D8), FTIR spectroscopy (PerkinElmer 1600 FTIR Spectrometer), Thermo Scientific K-Alpha™, X-ray Photoelectron Spectrometer (XPS) (Al Kα (1486.6 eV) and UV–vis measurements (6705 UV–vis JENWAY) were used to characterize the sensing materials.

An electronic digital balance (model: Scientech: ZSA 120) and a pH meter (sensION, SHA Snilu Instruments Co. Ltd., China) were used to weigh chemicals and measure the pH value of solutions.

A Metrohm potentiostat Autolab PGSTAT 20 (ECO CHEMIE Utrecht, The Netherlands) with software for multipurpose electrochemical systems (GPES) (North Carolina, USA) and frequency response analysis software (FRA) was used to perform the CVs and EIS analyses. For all electrochemical tests, a double-walled electrochemical cell with a standard three-electrode setup and a volume of approximately 40 ml was used.

The electrochemical analyses were carried out in 0.1 M (ammonium acetate). Within the potential range of −0.2 to 1V, the CVs, DPV characterizations were carried out. The electrochemical behavior of the samples was investigated by electrochemical impedance spectroscopy (EIS), and the impedance spectra were recorded at open-circuit voltage with an AC voltage amplitude of 10 mV in the AC frequency range of 100 kHz to 0.1 Hz.

## 3 Results and discussion

### 3.1 Characterizations of the sensing materials

#### 3.1.1 1.Morphological caracterisation

**Fig. 1:**
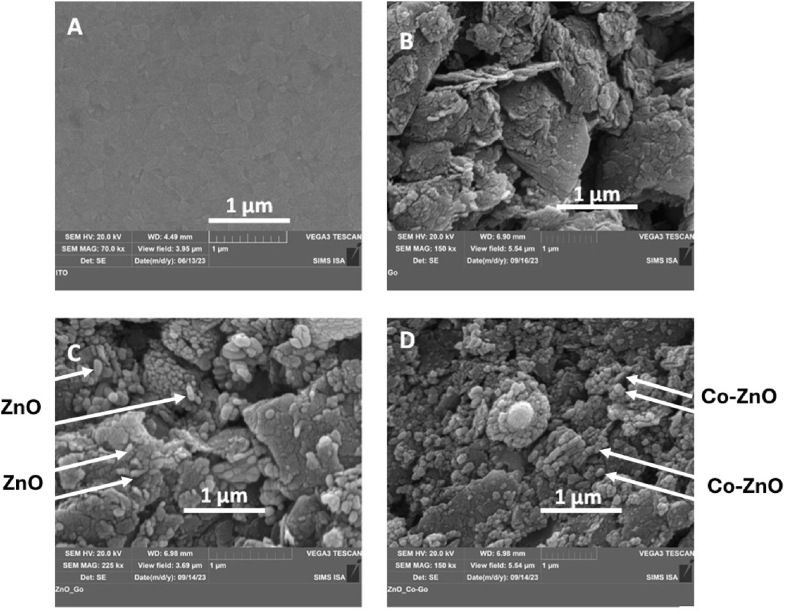
SEM of (A) ITO, (B) GO (C) ZnO/GO and (D) Co–ZnO/GO nanocomposite.

Surface morphologies of bare ITO, GO/ITO, ZnO/GO/ITO and Co–ZnO/GO/ITO electrodes were carried out by scanning electron microscopy (SEM). As shown in Fig. 1A, the bare ITO electrode presents a homogeneous and smooth surface. The morphology of the GO/ITO electrode is shown in Fig. 1B. It exhibits a wrinkled architecture and flake-like shapes, possibly caused by exfoliation. In the ZnO/GO/ITO electrode [Fig. 1C], many ZnO nanoparticles were seen as crystalline structures on the surface of the GO (length around 150 nm, diameter around 50 nm). This proves that ZnO/GO was successfully dispersed on the GO surface, at the electrode surface. The morphology of Co-doped ZnO/GO can be clearly seen in Fig. 1D. It shows a wide number of oblong structures, indicating that graphene provides an adequate surface area for the dispersion of Co–ZnO nanoparticles which then provide a larger specific surface area and thus more active sites available for the interaction with the analytes.

#### 3.1.2 Surface wettability

Wettability is an important property when the surface is used for sensor devices.

**Fig. 2:**
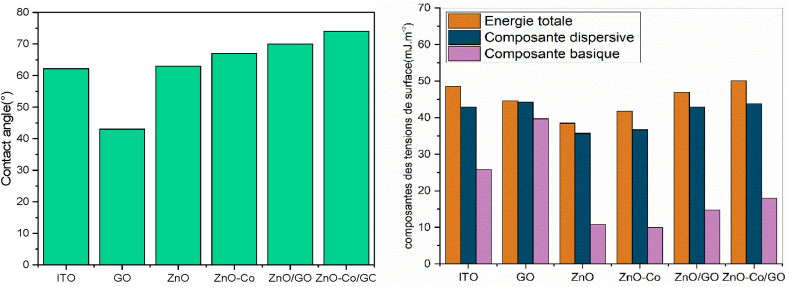
(A) Contact angles measurements, (B) Total Energy, Dispersive Component, Polar Component, Components of the surface tension.

To investigate the Co–ZnO/GO/ITO electrode fabrication, contact angle (CA) measurements were carried out using the sessile drop method. As shown in Fig. 2A, the CA value of bare ITO is (62° ± 1), which decreases to 43° ± 1 after the deposition of GO, may be due to the polar groups –COO- of GO that increase the hydrophobicity of the electrode surface [23]. A further increase in the contact angle with the immobilization of ZnO/GO nanocomposites (70° ± 1°) was observed. This result shows that the hydrophobicity of the membrane increases with the addition of ZnO nanoparticle in the GO matrix. After the functionalization process with the Co–ZnO/GO nanocomposite, the hydrophobicity has increased again (73.9° ± 1°). This hydrophobic behavior proves the presence of electrostatic interactions between the nanocomposite and the electrode. Thus, we can confirm the successful adhesion of the membranes to the ITO electrode surface.

The surface free energy's polar component, which indicated polar interactions, was changed into acid-base interactions according to the van Oss method [24].

As can be seen in Fig. 2B, the total free energy of ZnO and co-ZnO increases, while the dispersive component remains the same and the polar component decreases. However, for ZnO/GO and Co-doped ZnO/GO, the dispersive component remains constant, while the total free energy and the polar component increase. The polar components values indicate that there is an increase in the number of accessible surface sites on the Co-doped ZnO/GO surface designed for trace analyte detection.

#### 3.1.3 X-ray diffraction analysis

**Fig. 3:**
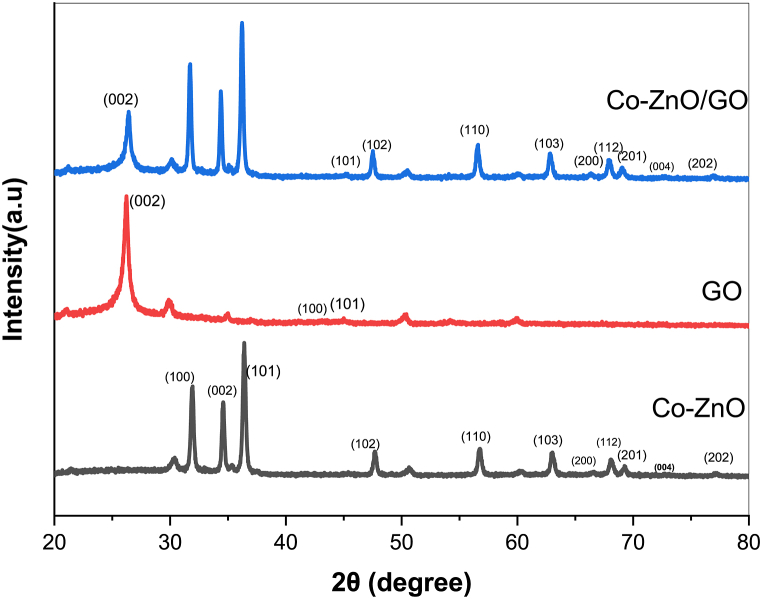
XRD analysis of Co–ZnO, GO and Co–ZnO/GO.

XRD patterns of cobalt doped- ZnO nanoparticles, GO and Co–ZnO/GO are shown in Fig. 3.

For Co– ZnO, the diffraction peaks in planes (100), (002), (101), (102), (110), (103), (200), (112), (201), (004) and (202) are all compatible with the hexagonal structure (wurtzite) JCPDS card N° 36–1451. The sample with GO showed three diffraction peaks with intensities of.

2θ = 24.66 (002), 42.57 (100) and 44.48° (101) indicating the graphite structure [25]**.**The XRD spectrum of Co–ZnO/GO nanocomposite shows the presence of co-doped ZnO peaks overlaying the surface of the GO lattice. The Crystallinity of the nanocomposite is very similar to that of co-doped ZnO, which indicates that graphene oxide provides a platform on which nanoparticles can be adsorbed [26]. Furthermore, the presence of the GO peak in the composites suggests that the nanoparticles could adhere to these graphene layers, which could prevent aggregation.

#### 3.1.4 Fourier transform infrared (FTIR) spectroscopy

**Fig. 4:**
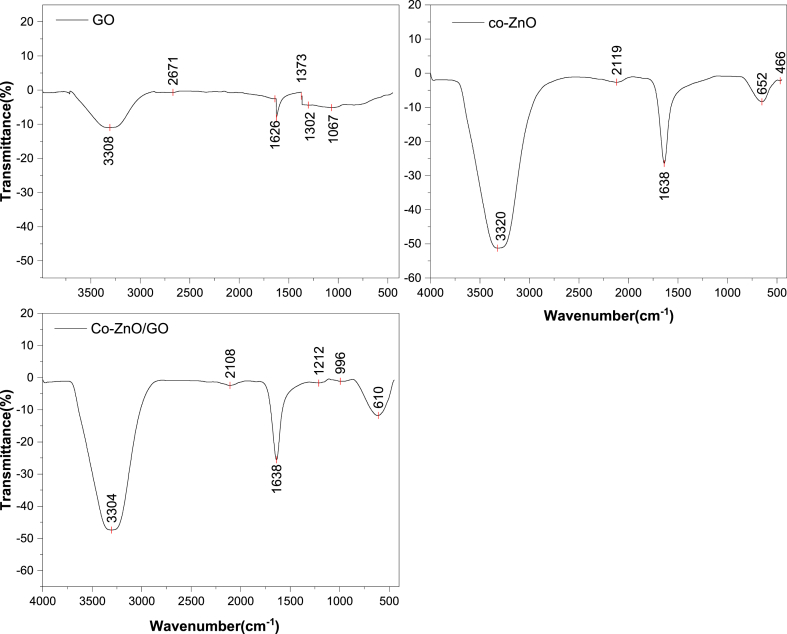
FTIR spectra of GO, Co–ZnO and Co–ZnO/GO.

To investigate the chemical bond structures of Graphene oxide, cobalt-doped ZnO, and co-ZnO/GO samples, FTIR spectroscopy was employed. As shown in Fig. 4, The FTIR spectra of (GO) exhibit distinct peaks at specific wavenumbers, 1040, 1617, 1726 and 3308 cm⁻^1^, corresponding to C─O stretching, sp^2^-hybridized C=C, C=O stretching bonds and OH stretching, respectively [ 27,28]. In Co–ZnO, broad absorption peaks are observed at 3332

cm^−1^ and 1638 cm^−1^, corresponding to the O–H stretching and H–*O*–H bending vibrations, respectively. These peaks indicate the presence of small amounts of H₂O content, probably as moisture, on the surface of the Co–ZnO nanocrystals [27,28].

Another band, observed between approximately 2100–2130 cm⁻^1^ corresponds to the vibration of acetate CH₂ (C–H) groups [29]. The band at 466 cm^−1^ corresponds to the stretching of oxygen metal (Zn–O) [30].A strong and sharp peak appeared at 652 cm^−1^ confirms the presence of cobalt stretching vibration [31,32].

For Co–ZnO/GO nanocomposite, the spectra shows the peaks at 1638 cm^−1^ corresponding to C=O stretching bond, which got shifted from 1626 cm^−1^ in case of pure GO due to composite formation. The other peaks observed in the range of 900–1400 cm^−1^ belongs to functional oxide groups of GO [33]. The peak at 1037 cm^−1^ was attributed to the presence of carbonyl group in the Co–ZnO/GO structure [34]. Thus FTIR results confirm the formation of Co–ZnO/GO nanocomposite.

#### 3.1.5 X-ray photoelectron spectroscopy (XPS) analysis

To confirm the formation of Co–ZnO/GO nanocomposite, X-ray photoelectron spectroscopy (XPS) analysis was used. It is a surface-sensitive analytical method that's used to identify the chemical composition of the surface.

**Fig. 5:**
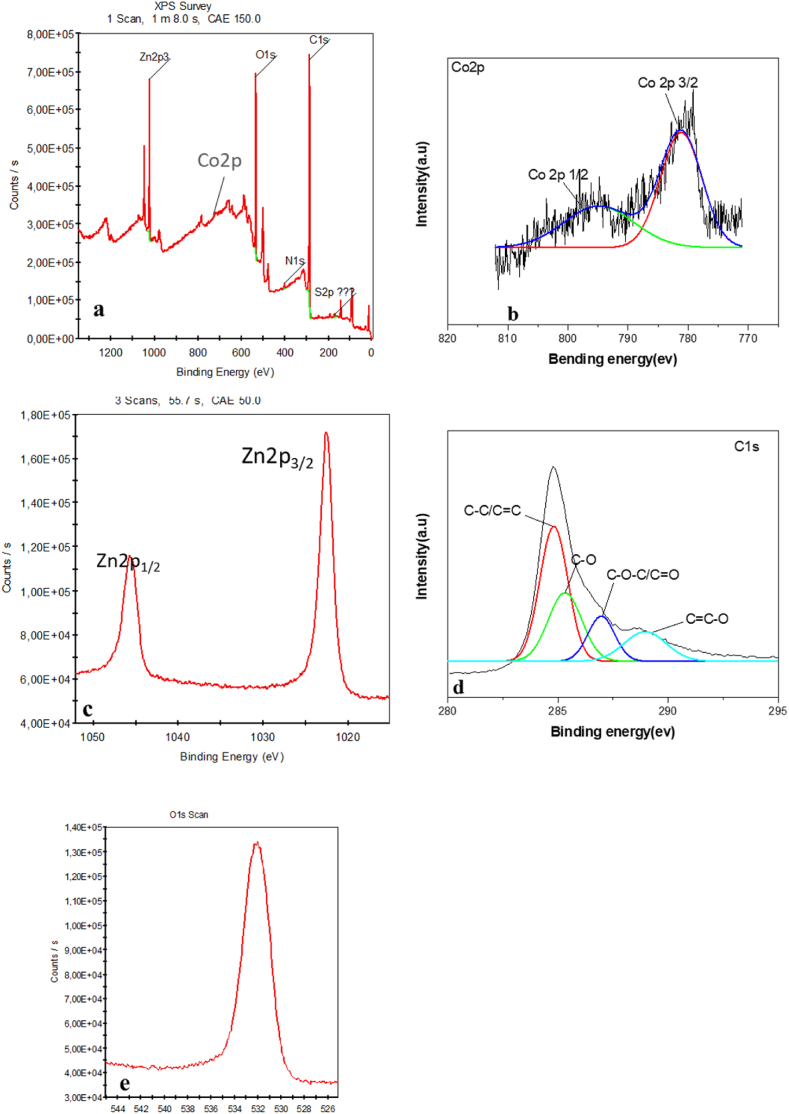
(a) XPS a survey spectrum of the prepared co-ZnO/GO sample (b) cobalt region; (c) Zn2p region; (d) C 1s region; (e) O1s region.

The Co–ZnO/GO substance's general spectrum, shown in Fig. 5a, clearly indicate the presence of Cobalt, Zinc, Oxygen, and Carbon in the material. The two peaks in Fig. 5 b, which are located at 795.21 and 781.16 eV, attributed to the electronic states of Co 2p ½ and Co 2p 3/2, respectively [35].

The Zn 2p3/2 and Zn 2p1/2 peaks, shown in Fig. 5c, are located at 1044.8 and 1021.8 eV, respectively. In Fig. 5d, the convoluted C 1s spectra can be subdivided into four peaks at 289.1, 286.3, 284.9, and 284.6 eV,which are identified as C=C–O, C–O–C/C=O, C–O, and C– C/C=C groups of GO, respectively [36].

Fig. 5e also shows the orbital spectrum of O 1s, located at 532.0 eV and identified as lattice oxygen presence in the nanocomposite [37].

The XPS analysis thus clearly confirms the existence of Co^2+^, Zn ^2+^, O^2−^, C=O and C–C in the nanocomposite produced.

#### 3.1.6 UV–visible absorbance analysis

**Fig. 6:**
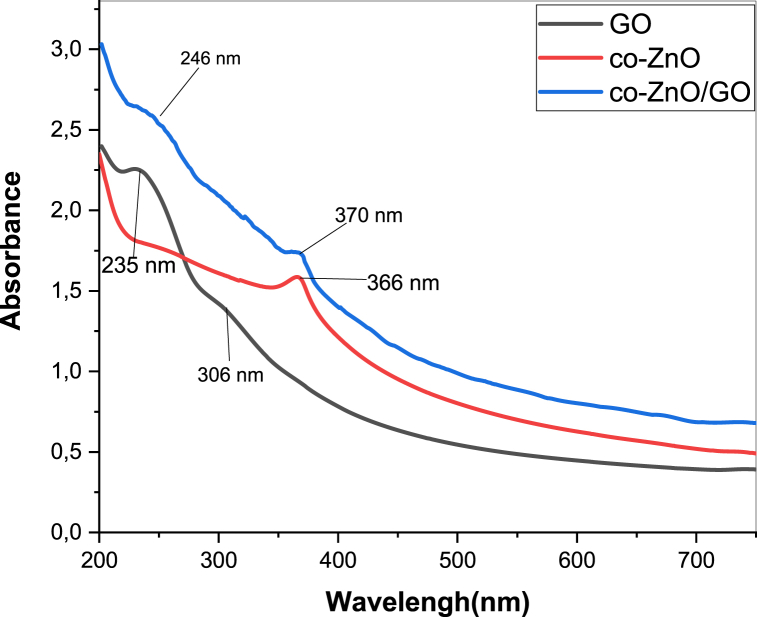
UV–vis absorbance spectra of GO, co-ZnO and co-ZnO/GO.

To analyze the optical properties of GO, Co–ZnO and Co–ZnO/GO nanomaterials**,** UV-VIS absorbance analysis was evaluated. As shown in Fig. 6, the GO shows a strong bands absorption peak at 235 nm and 306 nm, which is attributed to the π─π* transition of the aromatic C=C carbon and n–π* transition of the C=O band [38].

The absorption spectrum of Co–ZnO nanoparticles exhibits a broad band at 366 nm, which is related to the inherent band gap absorption of Co–ZnO [39]. Although the absorption spectra of the Co–ZnO/GO nanocomposite show one absorption peak at 246 nm associated with the GO layer present in the composite, and the other peak at 370 nm, which is associated with Co–ZnO.

A small red shift in the absorption peaks is observed due to the slight change in the nanostructure of Co–ZnO and GO nanomaterials.

### 3.2 Electrochemical analysis of the modified electrodes

**Fig. 7:**
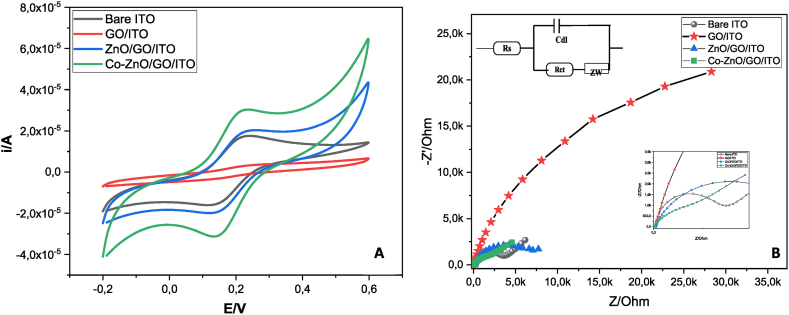
A (CV) and B (EIS) performed on bare ITO, GO/GCE, GO/ITO, ZnO/GO and Co–ZnO/GO/ITO in a 5.0 mM K_3_Fe(CN)_6_ solution and 0.1 M (CH_3_COO^−^, NH_4_^+^)at the scan of 0.05V s^−1^..

The electrochemical properties of bare ITO and its modified forms of electrodes were evaluated using cyclic voltammetry and electrochemical impedance spectroscopy in a solution of 0.1 M KCl that contains 5 mM K_3_[Fe(CN)_6_] which was used as a marker to assess the changes in the electrode behavior following each modifying process (Fig. 7).

From Fig. 7A, it can be observed that a typical redox reaction takes place at - 0.2 V–0.6 V for the bare ITO electrode. The GO deposition reduced significantly the response current of [Fe (CN) 6]^3−/4−^ with a peak potential anodic (Epa) at 0.26 V and cathodic (Epc) at 0.12V due to the poor conductivity of the graphene oxide and the small surface area. Furthermore, after deposition of ZnO/GO nanocomposite, there is an unprecedented increase in current peaks around 7-fold approximately as compared to pure GO and the peak potentials were shifted to negative value of anodic peaks at 0.24V and positive value of cathodic peak at 0.14 V.

This increase is due to the combination of ZnO nanoparticles and the GO which can increase the surface area and the conductivity.

Consequently, a distinct and well-defined redox peak (Epa = 0.22V, Epc = 0.14V) with the largest anodic and cathodic peak currents was obtained for the Co–ZnO/GO/ITO. Besides, these results indicate that doping cobalt greatly improved electrochemical performances of ZnO/GO.

Using the Randles-Sevcik formula the electrochemical activity area can be determined as follows [39,40]:

(2) I_pc_ = 2.691 × 10^5^n^3/2^D^1/2^V^1/2^AC

in which Ipc is the K_3_Fe (CN)_6_ peak current, n is the number of electrons transported during the electrochemical reactions, D is the diffusion coefficient of K_3_Fe (CN)_6_ (D = 7.6 10^−6^cm^2^s^−1^), V is the scan rate (V/s), A is the electrochemical activity area (cm^2^) and C is the concentration of K_3_Fe(CN)_6_ (mol/cm^3^). Hence, the electrochemical active areas of bare ITO, GO/ITO, ZnO/GO/ITO and Co–ZnO/GO/ITO can be calculated as 0.019, 0.004, 0.024 and 0.047 cm^2^, respectively. These studies clearly prove that Co doping ZnO significantly enhances both the surface area and the electron transport property, providing ample active sites for the adsorption of antibiotic molecules. This increase in surface area enhances the sensitivity of the electrochemical sensor, which make Co-doped ZnO/GO based sensors promising candidates for sensitive, selective, and reliable detection of oxytetracycline antibiotics in various real-world applications.

To validate the results of the CV experiments, electrochemical impedance spectroscopy (EIS) investigations were also carried out. This technique has been widely utilized to examine the interfacial properties of different electrochemical sensors [41]. As shown in Fig. 7B, Nyquist graphs are semicircular at higher frequencies in relation to the electron transfer-limited mechanism. The diameter of the semicircle represents the charge transfer resistance (R_ct_).

All of the spectra were fitted using the Randles circuit model shown in the inset of Fig. 7B.This model involves the charge transfer resistance (R_ct_),the electrolyte solution resistance (R_s_),the double layer capacitance(C_dl_) and the Warburg element's impedance(Zw).

The R_ct_ values are 3697, 19073, 5574, 1554 Ω respectively, for the bare ITO, GO/ITO, ZnO/GO/ITO, and Co–ZnO/GO/ITO. Herein, we demonstrate that when ITO was modified with Co–ZnO/GO, the R_ct considerably_ decreased, which can be attributed to the higher electro-conductivity, as it was demonstrated in Ref. [20] that the presence of Co decreases the band gap width, and leads to a higher specific surface area of Co–ZnO/GO.

### 3.3 Electrochemical behavior of oxytetracycline on various electrodes

**Fig. 8:**
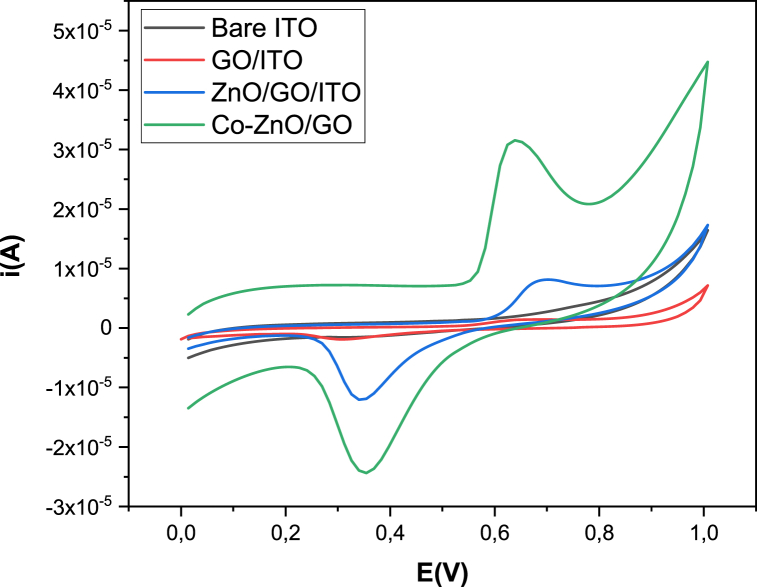
Electrochemical behavior of 10 μM OTC on the bare ITO (a), GO/ITO(b), ZnO/GO/ITO (c) and Co–ZnO/GO/ITO (d) in 0.1 M ammonium acetate (CH_3_COO^−^, NH_4_^+^)) (pH 7.0) at a scan rate of 50 mV ^−1^.

Fig. 8 illustrates CV responses of GO/ITO, ZnO/GO/ITO and Co–ZnO/GO/ITO in 0.1 M ammonium acetate (pH = 7). A very low peak current appeared after GO modification, indicating the poor electrocatalytic activity of GO/ITO towards OTC detection. After incorporating the ZnO nanomaterials into the electrode matrix, the electrochemical peak currents, either the oxidation or the reduction peaks, increased. Co–ZnO/GO nanocomposite exhibited the highest electrochemical signal. The oxidation and reduction peak currents of OTC were obtained at 0.65V and 0.35V, respectively, without any addition of an artificial redox mediator. It appears that Co–ZnO/GO nanocomposite offers a large specific surface area and a higher electrocatalytic activity. It has greatly enhanced the electron transfer, which is induced by the doping cobalt into the ZnO matrix. These results prove that the Co–ZnO/GO/ITO is the adequate choice for determination of oxytetracycline.

### 3.4 Parameters affecting OTC determination

#### 3.4.1 Influence of scan rate

**Fig. 9:**
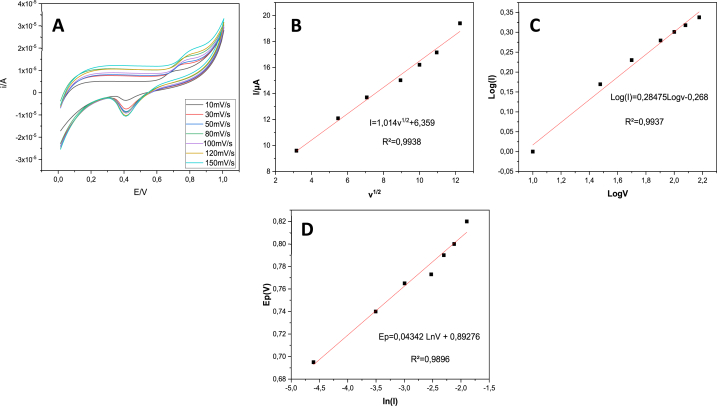
(A) CVs of 10 μM OTC at different scanning rates. (B) Linear relationship between the redox peak currents of OTC and square root of scan. (C) the relationship between log (ipa) and log (v). (D) Relationship between the oxidation peak potential (Ep) and the decimal logarithm of scan rate (lnv).

In order to evaluate the electrochemical process of OTC at Co–ZnO/GO modified ITO electrode, the influence of scan rate on the electrochemical oxidation of oxytetracycline was investigated using cyclic voltammetry method at different scan rates (10–100 mVs^−1^) (Fig. 9A).

As shown in Fig. 9B, the anodic current was increased linearly with an increase in scan root according to a regression equation:

(3) I = 1.014 v^1/2^+6.359, R^2^ = 0.9938

Also, the relationship between log (Ipa) and log (v) was plotted (Fig. 9C), satisfying the equation:

(4) Log (I) = 0.28475Logv-0.268, R^2^ = 0.99

The slope value of 0.26 provides the ideal reaction mechanism for a diffusion-controlled process [42]**.**

According to the slope value, the reaction mechanism is controlled by diffusion.

For such a quasi-reversible or irreversible reaction, the number of electrons (n) implicated in an oxidation reaction was determined using the Laviron's equation [43] as shown below:

(5) $$\text{Ep}=E{}^{\circ}+\left(\frac{\text{RT}}{\alpha \text{nF}}\right)\mathrm{Ln}\left(\frac{\text{RTK}{}^{\circ}}{\alpha \text{nF}}\right)+\left(\frac{\text{RT}}{\alpha \text{nF}}\right)\text{Lnv}$$

Where E° is the standard potential, α is the electron transfer coefficient of the oxidation of oxytetracycline, n is the number of electrons participating in the redox process, F is the Faraday constant (F = 96485 C mol^−1^), R is the ideal gas constant (8.314 J K^−1^mol^−1^) and (T = 298.15K).

Moreover, a linear relationship between potential and Lnv was obtained (Fig. 9D):

(6) Ep = 0.04342 Lnv +0.89276, R^2^ = 0.99

(αn) can be calculated as 0.597 using equation (2) and the linear equation $\frac{dEp}{Lnv}$ = $\frac{RT}{\propto nF}$ = 0.043. Generally, α is assumed to be 0.5 for an irreversible process. Therefore, n can be calculated as 1.19. The number of electrons in the OTC oxidation reaction was therefore calculated to be 1, in agreement with earlier studies [[44], [45]].

#### 3.4.2 Effect of pH

**Fig. 10:**
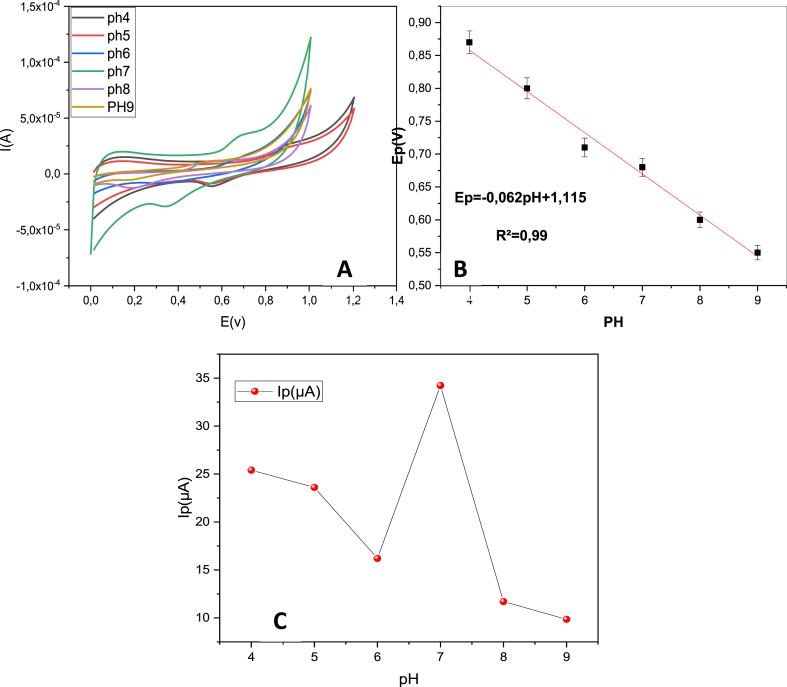
(A) CV pattern of 10 μM OTC on Co–ZnO/GO/ITO at different pH; (B) the linear relationship of the anode peak potential of OTC and the pH; (C) the effect of pH on the current of oxytetracycline in 0.1 M (CH_3_COO^−^, NH_4_^+^).

The electrolyte's pH can affect both, the peak potential and peak current of the modified electrode in the presence of analyte. Moreover, it is crucial in estimating the ratio proton to electron involved in the electrode reaction. As shown in Fig. 10, the effect of pH was studied

from 4 to 9 at a scan rate of 50 mV s^−1^. It is clear to observe, in Fig. 10A, the negative shift of peak potential with the increase of pH values, confirming the direct participation of protons (H^+^) in the rate-controlling step [46].

Fig. 10B shows the relationship between Ep and pH with a linear regression of

(7) Ep = **-**0.0638 pH + 1.115, R^2^ = 0.9831

**Scheme 1:**
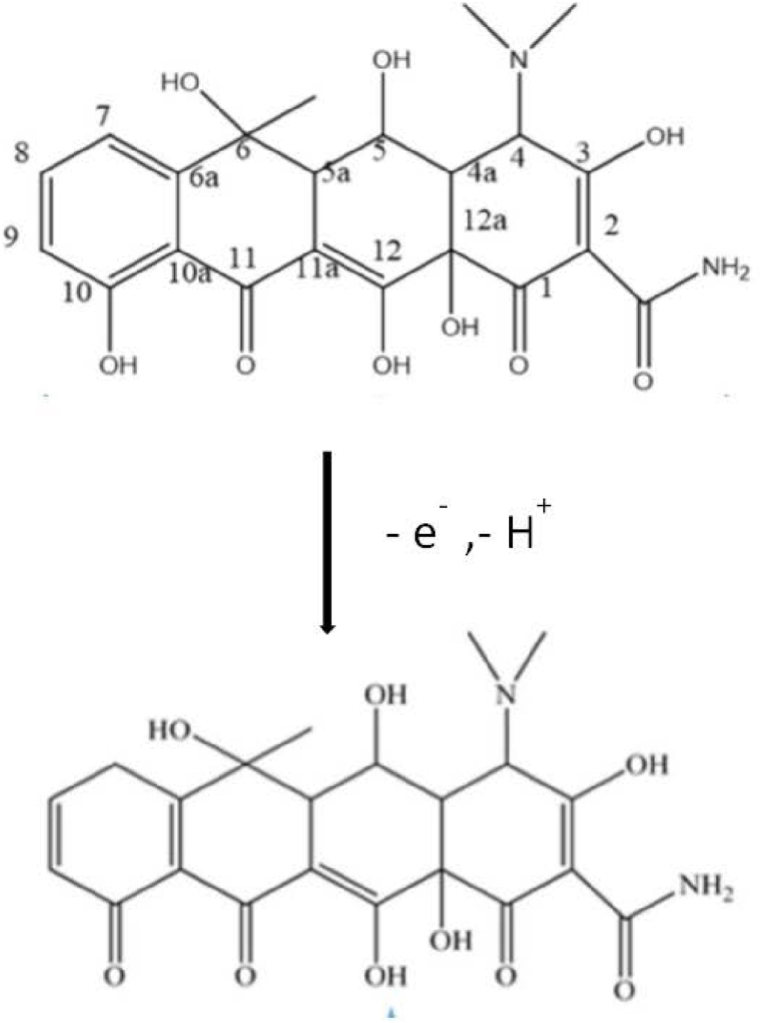
Mechanism of detection of oxytetracycline.

Compared to the theoretical value of 59.2 mV pH^−1^ at 25 °C, the slope value of 62.8 mV pH-1 is very similar, which suggests that the OTC oxidation reaction involved a ratio of 1 electrons to protons. The possible mechanism for the electrochemical reactions at the electrode surface is shown in Scheme 1 [46,47].

As shown in Fig. 10C, The oxidation current decreased gradually from 4 to 6, where the concentration of protons (H^+^) is relatively high. These protons can interact with the electrode surface and affect the kinetics of the oxidation reaction. Then become maximum at pH 7 which can be attributed to the balance between proton concentration and the activity of the oxytetracycline. At this pH, the electrochemical reaction kinetics are favored, leading to efficient oxidation of the analyte at the electrode surface and consequently maximizing the current response. After pH7, the concentration of OH^−^ ions increases, which can affect the surface chemistry of the electrode the presence of excess hydroxide ions may lead to the formation of insoluble hydroxide precipitates or other side reactions that compete with the oxytetracycline oxidation process, resulting in a decrease in the oxidation current.

The best response was observed at pH 7, hence, for an analytical perspective, a neutral pH was chosen for further electrochemical determination of oxytetracycline.

#### 3.4.3 Influence of accumulation parameters

Reaction peak currents can be effectively enhanced by the accumulation of the target species, so the effects of accumulation potential and time were also investigated.

**Fig. 11:**
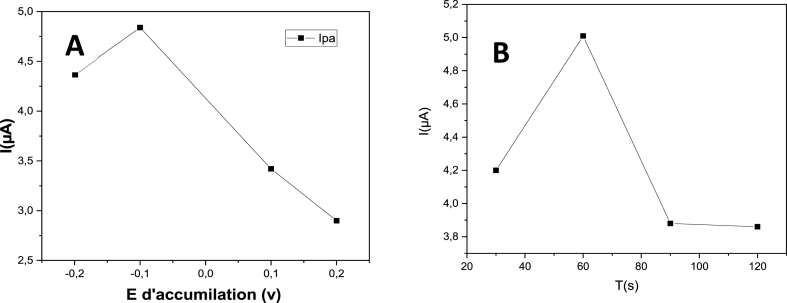
(A) Deposition potential and (B) deposition time on the oxidation peak currents of 10 μM oxytetracycline (0.1 M (CH_3_COO^−^, NH_4_^+^), pH = 7) at Co–ZnO/GO/ITO.

The peak currents of OTC sharply increased with the accumulation potentials shifting from −0.2 V to −0.1 V, then gradually decreased with a further increase of the accumulation potential (Fig. 11A). The highest anodic peak current of OTC was achieved at −0.1 V. Thus, this potential was chosen as the optimal accumulation potential.

As illustrated in Fig. 11B, the anodic peak current increased progressively during the first 60 s, then decreased with the prolongation of the accumulation time. As a result, in the following tests, accumulation was carried out for 60 s at −0.1 V.

### 3.5 Electrochemical determination of oxytetracycline

**Fig. 12:**
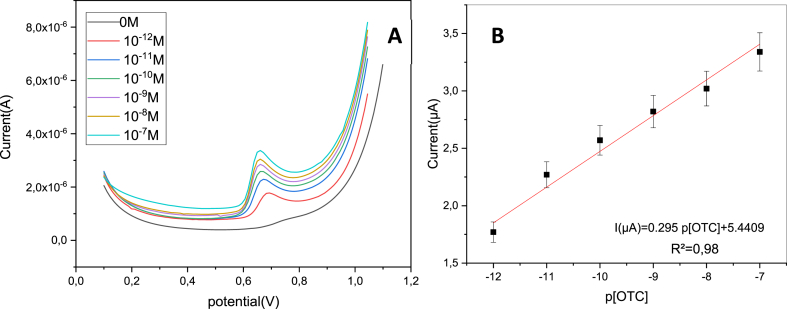
(A) DPV obtained on CO–ZnO/GO/ITO in 0.1 M (CH_3_COO^−^, NH_4_^+^) (pH 7) containing different concentrations of oxytetracycline (OTC) (10–12 to 10-7). (B) The linear relationship between the anodic peak currents (ipa) of OTC and OTC concentration (OTC).

The main objective of this study is to employ the Co–ZnO/GO modified ITO electrode to make possible direct detection of OTC. In this regard, different concentrations of oxytetracycline were added into the electrochemical cell. The differential pulse voltammetry (DPV) measurements were performed versus ECS under the optimum lab conditions (potential range: 0–1 V, scan rate = 50 mVs-1^,^ and step potential = 0.01 V). As shown in Fig. 12A, the oxidation peak current increased with oxytetracycline concentration, indicating the rapid electron transfer and the electrocatalytic efficacy of the fabricated nanostructured electrode. Fig. 12B shows the corresponding calibration curve that has a linear response between the peak current and the log of OTC concentration, from 10^−12^ to 10^−7^ M according to the following equation.

(8) I (μA) = 0.295 p [OTC] + 5.44 (R^2^ = 0.986)

The LOD and sensibility are determined from the calibration plot's slope, employing Eq. (9) and Eq (10).

(9) LOD = 3***σ***∕S

(10) Sensitivity = S/A

Where S is the slope of the calibration curve, A is the electrode's surface area, and ***σ*** is the standard deviation of blank runs. Thus, a good sensitivity (0.295) of Co–ZnO/GO/ITO can be obtained, and the limit of detection (LOD) (S/N = 3) is estimated as 1.610^−13^ M.When comparing the analytical performance of the developed electrochemical sensor for detecting oxytetracycline (OTC) with previously reported studies, our sensor demonstrated promising results in terms of sensitivity, selectivity, and real sample application (Table 1).

**Table 1:** Comparison between the analytical parameters of the proposed sensor with the other reported methods for OTC electrochemical determination.

Electrode	Electrochemical technique	Linear range	Limit of detection	References
Ta2O5-ErGO/GCE	CV	0.2–100 μM	0.095 μM	[48]
C-doped ZnO	PEC	0.01–1000 nM	8.75 pM	[49]
DPA@CuNCs	Fluorescence	5–60 μM	0.026 μM	[50]
AuNP@MIPs-CdTe QDs	Fluorescence	0.1–3.0 μM	5.22 nM	[51]
MWCNTs-AuNPs/CS-AuNPs/rGO-AuNPs	DPV	1–540 nM.	30pM	[52]
EPPU/GCE	SWV	1–200 μM	1.01 × 10^−2^ μM	[53]
B-CDs	FL	0.33 μg/mL.	1.52–27.60 μg/mL	[54]
EDCA [4]	SIE	2.3 × 10^−11^ M.	10^−10^ M to 10^−5^ M	[54]
Co–ZnO/GO/ITO	DPV	1 pM to 0.1 μM	0.16 pM	Our work

The stability of the OTC sensor was studied after storage in a refrigerator at 4 °C for one month; the catalytic current response was maintained at 91 % of its initial value after one month.

### 3.6 Interference analysis

**Fig. 13:**
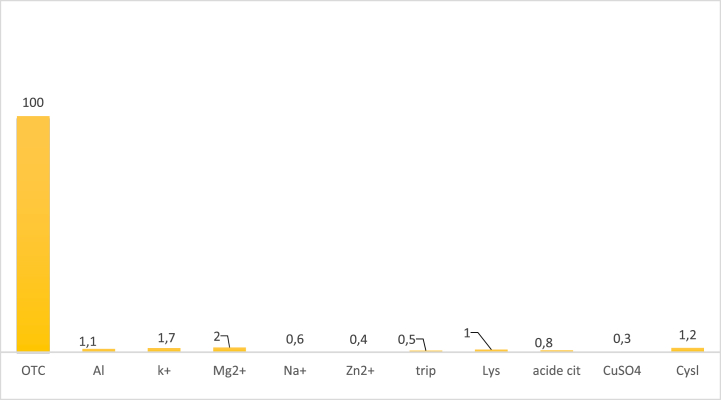
Relative response of the OTC sensor for some species at pH = 7.

The selectivity performance of the OTC sensor was evaluated under optimal analytical conditions. The reactions of some inorganic ions (K^+^, Na^+^, Zn^2+^, Cu^2+^, Mg^2+^, Al^3+^) and organic compounds (citric acid, tryptophan, cysteine, lysine) on the OTC sensor were investigated using DPV. As can be seen in Fig. 13, the response of the tested species is weak compared to that of 10 μM oxytetracycline (<3 %). These results show that the proposed Co–ZnO/GO/ITO sensor has a high selectivity in the determination of OTC.

### 3.7 Analytical application

**Fig. 14:**
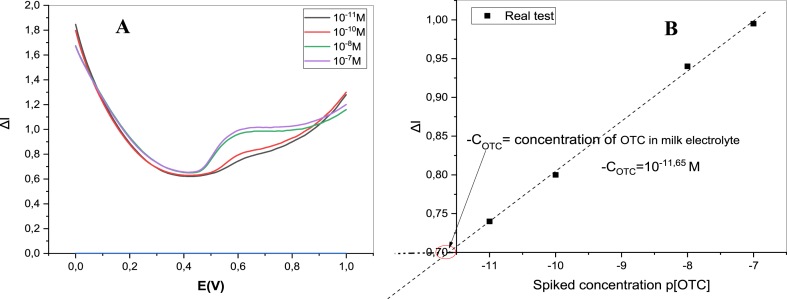
DPV responses of the Co–ZnO/GO/ITO for the real detection of OTC in the presence of four concentration of OTC in milk electrolyte solution, (B) Calibration curve obtained by standard addition method.

In order to evaluate the practical usability of the novel Co–ZnO/GO/ITO sensor, a diluted milk sample was tested in 0.1 M ammonium acetate (pH = 7) as a supporting electrolyte by the DPV technique. The Standard addition method was chosen to determine the total amount of OTC in the milk electrolyte solution and the results are displayed in Fig. 14. Therefore, the milk sample was spiked with four concentration of OTC (0.01, 0.1,10, and 100 nM). As shown in Fig. 14B, the initial concentration of OTC in the milk electrolyte was determined to be around 10^−11.65^M [55].For calculating the recovery percentage (%), the following equation was used: Recovery% = 100 (C_Found_−C_initial_)/C_added,_ where C_Found_ is the concentration of the total OTC found in the sample after spiking, C_initial_ is the amount of OTC in original sample and C_added_ is the concentration of OTC spiked. As shown in Table 2, the recovery of OTC was in the range of **98.83 % and 108.4 %** which suggests that there were no appreciable interferences from the sample's matrix. These results demonstrate that the fabricated Co–ZnO/GO/ITO-based sensor has excellent potential for use in oxytetracycline detection in real samples, such as milk matrices.

**Table 2:** OTC in the milk supernatant sample by DPV (n = 3).

Sample	*C*_*initial in*_*Milk electrolyte solution (nM)*	*C*_*Added*_*(nM)*	*C*_*Found*_*(nM)*	*Recovery (%)*
		0.01	0.014	108.4
Milk	0.003162	0.1	0.102	98.83
		10	10.022	99.96
		100	100.014	100.01

## 4 Conclusion

In this work, we have reported a novel, simple and economical approach for the fabrication of cobalt-doped ZnO NPs on the GO surface. The resulting Co–ZnO/GO nanocomposite was deposited onto the ITO electrode. XRD, XPS, FTIR results confirm the formation of Co–ZnO/GO nanocomposite. The electrochemical measurements (CV and EIS) and the contact angle measurements confirmed that Co–ZnO/GO had been effectively deposited onto the surface of ITO electrodes. SEM measurements showed that many Co–ZnO nanoparticles were dispersed on the surface of GO which was not the case for ZnO/GO.

The obtained results revealed that the developed nanocomposite has great promise for an oxytetracycline electrochemical sensor. It exhibited an ultrasensitive detection for oxytetracycline with wide linear concentration range of 10^−12^ M to 10^−7^ M and a lower detection limit of 1.6 10^−13^ M. This OTC sensor presents an excellent potential for OTC detection in animal-derived foods such as milk.

## Data availability

Data will be made available on request.

## CRediT authorship contribution statement

**Haifa Mliki:** Writing – original draft, Software, Methodology, Investigation, Funding acquisition, Formal analysis, Data curation, Conceptualization. **Mosaab Echabaane:** Visualization, Investigation. **Ahlem Rouis:** Supervision. **Jaber Mohamed El Ghoul:** Methodology. **Francois Bessueille:** Software. **Dhekra Ayed:** Visualization. **Nicole Jaffrezic-Renault:** Supervision.

## Declaration of competing interest

The authors whose names are listed immediately below certify that they have NO affiliations with or involvement in any organization or entity with any financial interest (such as honoraria; educational grants; participation in speakers’ bureaus; membership, employment, consultancies, stock ownership, or other equity interest; and expert testimony or patent-licensing arrangements), or non-financial interest (such as personal or professional relationships, affiliations, knowledge or beliefs) in the subject matter or materials discussed in this manuscript.
